# Genetically engineered multicistronic allele of *Pmel* yielding highly specific CreERT2‐mediated recombination in the melanocyte lineage

**DOI:** 10.1111/pcmr.13076

**Published:** 2022-12-19

**Authors:** Emma L. Wilkinson, Louise C. Brennan, Olivia J. Harrison, Zoe Crane‐Smith, Philippe Gautier, Margaret A. Keighren, Peter Budd, Karthic Swaminathan, Laura M. Machesky, Sarah L. Allinson, Ian J. Jackson, Richard L. Mort

**Affiliations:** ^1^ Division of Biomedical and Life Sciences, Faculty of Health and Medicine Lancaster University Lancaster UK; ^2^ MRC Human Genetics Unit, Institute of Genetics and Cancer, Western General Hospital University of Edinburgh Edinburgh UK; ^3^ Centre for Skin Sciences, Faculty of Life Sciences University of Bradford Bradford UK; ^4^ Cancer Research UK, Beatson Institute, and Institute of Cancer Sciences University of Glasgow Glasgow UK; ^5^ Roslin Institute University of Edinburgh Roslin UK

**Keywords:** keratinocytes, melanoblasts, melanocytes, melanoma

## Abstract

Genetic approaches that allow lineage tracing are essential to our future understanding of melanocytes and melanoma. To date, the approaches used to label melanocytes in mice have relied on random integration of transgenes driven by the promoters of the *Tyrosinase* and *Dopachrome tautomerase* genes, knock‐in to the *Dopachrome tautomerase* locus or knock‐in to the *Mlana* locus in a bacterial artificial chromosome. These strategies result in expression in other tissues such as telencephalon and other cell types such as nerves. Here we used homologous recombination in mouse embryonic stem cells to generate a targeted multicistronic allele of the *Pmel* locus that drives melanocyte‐specific expression of CreERT2, nuclear localised H2B‐Cerulean and membrane localised marcks‐mKate2 allowing live imaging of melanocytes and activation of other conditional alleles. We combined this allele with *R26R‐EYFP* mice allowing induction of EYFP expression on administration of tamoxifen or its metabolite 4‐OHT. The fluorescent proteins H2B‐Cerulean and marcks‐mKate2 label the cell nucleus and plasma membrane respectively allowing live imaging and FACS isolation of melanoblasts and melanocytes as well as serving to provide an internal control allowing estimation of recombination efficiency after administration of tamoxifen. We demonstrate the utility of the transgene in embryonic and adult tissues.

**Significance**

Here we demonstrate the feasibility of using the mouse *Pmel* locus to drive melanocyte‐specific transgene expression without altering endogenous *Pmel* expression. The targeting strategy used would be easy to repeat with other genetic constructs to yield reproducible results not possible using random transgenesis.

In mouse, melanoblast precursors are thought to delaminate from the neural tube at embryonic (E) day 9 and upregulate melanocytic markers (*Mitf, Dct, Pmel*) at E10.5 concomitant with their entry onto the dorsolateral migratory pathway (Mort et al., 2015). At E12.5 a second melanoblast population is thought to arise from Schwann cell precursors and interdigitate with this early migrating population as they move from the dermis to the epidermis (Adameyko et al., 2009). At E15.5, melanocytes enter the developing hair follicles and later make a fate decision either differentiating into pigment producing melanocytes in the hair matrix or melanocyte stem cells (MSCs) in the hair follicle bulge. Epidermal melanocytes persist in mouse in the tail and the pinna of the ear (Mort et al., 2015). As the field builds its understanding of how melanoblast behaviour is regulated during colonisation of the developing embryo it increasingly relies on genetic labelling, live‐cell imaging and targeted knock‐out of key effector genes (Li et al., 2011; Mackenzie et al., 1997; Mort et al., 2016).

In order to label and genetically manipulate melanoblasts and melanocytes, transgenic strategies have been developed that rely on driving Cre recombinase or Cre recombinase fused to the oestrogen receptor (CreERT2) in a spatially restricted manner. These strategies have either: (1) used a promoter fragment from the *Tyr* (Bosenberg et al., 2006; Delmas et al., 2003; Yajima et al., 2006) or *Dct* genes randomly integrated into the genome (Davis et al., 2009); (2) have targeted the endogenous *Dct* locus (Davis et al., 2009; Guyonneau et al., 2002) or; (3) have targeted the *Mlana* locus contained within a bacterial artificial chromosome (BAC) which was then randomly integrated into the genome (Aydin & Beermann, 2011). The activity of these Cre ‘drivers’ can be monitored by using a ‘reporter’ strain such as *R26R‐EYFP* which is activated by Cre‐mediated recombination of loxP sites flanking a transcriptional stop sequence (Mort et al., 2010; Srinivas et al., 2001). Other transgenes or targeted alleles can be recombined at the same time allowing activation of aberrant signalling events or ablation of essential genes – for example to induce melanoma (Viros et al., 2014). However the existing transgenic models have several limitations.

First, driver strains that rely on random genomic integration are prone to position effects and copy number variation which result in expression patterns that do not exactly mirror the endogenous promoter activity and that vary unpredictably between lines (al‐Shawi et al., 1990; Wilson et al., 1990). Second, the *Dct* and *Tyr* promoter fragments generally used are active in other tissues such as the telencephalon and ectopically in other cell types such as the dorsal root ganglia, craniofacial structures, sympathetic cephalic ganglia and caudal nerves (Guyonneau et al., 2002; Mackenzie et al., 1997; Tonks et al., 2003). This makes knock‐out of essential genes difficult because of the unintended consequences of ablating them in these tissues and makes applications that require pure populations such as live imaging and single cell RNAseq challenging. Third, when driving CreERT2 (the preferred Cre variant for lineage tracing) it is difficult to judge the recombination efficiency in melanocytes because none of the driver strains label melanoblasts or melanocytes directly. Here we set out to develop a new transgenic model that combines highly specific melanocytic expression of CreERT2 with fluorescent probes that label melanocytes directly for imaging studies.

*Pmel* is an MITF‐dependent melanoblast and melanocyte‐specific gene that demonstrates robust expression preceding *Dct* in dorsal regions at around E10.5 (Baxter & Pavan, 2003). In order to develop a flexible and highly melanocyte‐specific CreERT2 mouse line for live‐imaging experiments we set out to address the limitations of existing melanocyte‐specific Cre and CreERT2 driver strains by knocking our construct into the endogenous *Pmel* locus (see Appendix S1 for detailed methods). First, we built a live‐imaging cassette that combined CreERT2 (C) with a membrane targeted marcks‐mKate2 fluorescent marker (M) and a nuclear localised H2B‐Cerulean marker (N) in a tricistronic construct (CMN) and tested its functionality in HeLa cells (Figure 1a,b). Next we built a *Pmel* targeting construct by fusing the CMN cassette to Exon11 of the *Pmel* gene using a foot and mouth disease virus 2a peptide sequence (f2a), incorporating an frt flanked PGK‐Neo selection cassette and flanking the construct with homology arms amplified by PCR (Figure 1c). The construct was incorporated into mouse embryonic stem cells by homologous recombination and faithful insertion into the endogenous locus was confirmed by long‐range PCR across the homology arms (Figure 1d) followed by breeding to homozygosity (Figure 1e). The PGK‐Neo selection cassette was later removed by crossing with FLPe‐deleter mice (Rodríguez et al., 2000). *Pmel* ^*CMN/CMN*^ were viable and we did not observe changes to the coat colour phenotype in these animals.

**FIGURE 1:**
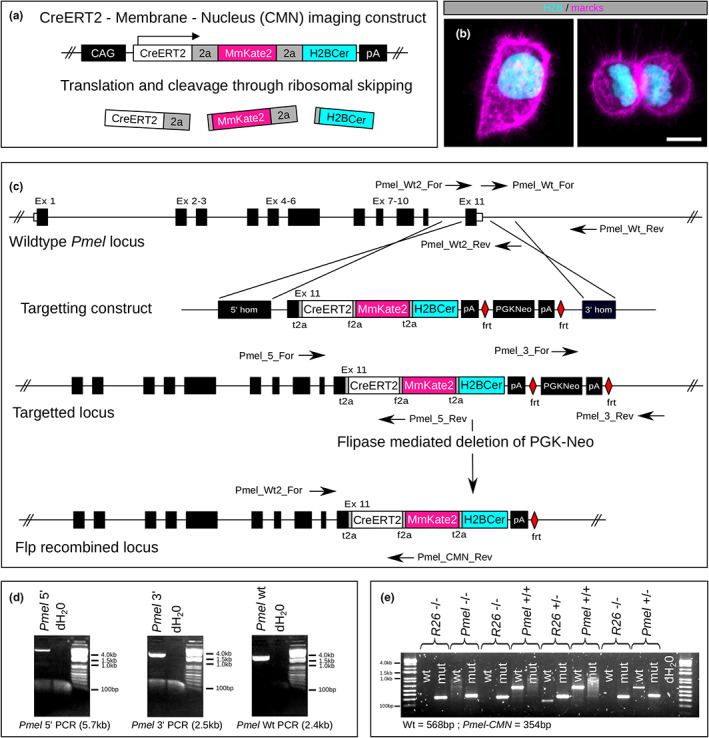
Construct design and targeting of the *Pmel* locus in MESCs. (a). the CreERT2, membrane and nucleus (CMN) imaging construct was made by fusion of CreERT2 by self cleaving peptide to a marcks‐mKate2 (MmKate2) membrane‐localised probe and H2B‐Cerulean (H2BCer) nuclear localised probe driven by the CAG promoter for testing. (b). Transient transfection into HeLa cells confirmed correct localisation of marcks‐mKate2 and H2B‐Cerulean. (c). To target the *Pmel* locus Exon11 of *Pmel* was fused by self‐cleaving peptide to the CMN cassette followed by an Frt‐flanked PGK‐Neo selection cassette. The whole construct was then flanked by 5′ and 3′ homology sequences cloned by PCR from the *Pmel* locus with a 5′ breakpoint in *Pmel* intron 10. The construct was incorporated into the *Pmel* genomic locus in E14 mouse embryonic stem cells (MESC) by homologous recombination and mice generated by standard blastocyst injection. The PGK‐Neo selection cassette was removed by crossing with FLPe‐deleter mice (JAX:003800). (d). Long range PCR on a correctly targeted MESC clone confirming the integrity of the wildtype allele (*Pmel* wt) and the correct integration of the construct by PCR across the 5′ and 3′ homology arms (*Pmel* 5′ and *Pmel* 3′). (e). Genotyping of mice from a *Pmel‐CMN;R26‐EYFP* cross. Identification of littermates homozygous and heterozygous for the targeted and wildtype *Pmel* alleles confirmed faithful integration into the genomic locus. f2a, foot‐and‐mouth disease virus 2a peptide; t2a, thosea asigna virus 2A peptide; wt, wild type; Mut, mutant; MmKate2, marcks‐mKate2. Scale bars in B = 10 μm.

In order to assess the activity of CreERT2 in our *Pmel‐CMN* mouse line, we crossed it with the *R26R‐EYFP* reporter line (Figure 2a) and administered a single Tamoxifen or 4‐OHT dose to pregnant dams at E10.5 or E11.5. We observed expression of marcks‐mKate2 and H2B‐Cerulean in melanoblasts in the trunk, the limb, the head and the retinal pigmented epithelium at E13.5 and E14.5 (Figure 2b–d). Although the levels of H2B‐Cerulean always appeared robust in embryos it was sometimes hard to identify melanoblasts using marcks‐mKate2 alone (Figure 2b,c). This was presumably because while the histone tag could accumulate in the nuclei of melanoblasts the plasma membrane is highly dynamic. Only a subset of melanoblasts were labelled with EYFP, indicating that recombination is not induced in all *Pmel* expressing cells with a single dose of either Tamoxifen or 4‐OHT. In time‐lapse movies we could clearly observe cell migration, pseudopod dynamics, and progression through mitosis and this was particularly well delineated when viewing the three fluorescent tags (Video S1). The *Pmel* ^*CMN/CMN*^ *; Rosa26* ^*EYFP/EYFP*^ model therefore allows tracking of cells in which CreERT2 has been activated as well as visualising all cells in which *Pmel* is transcriptionally active allowing one to track cells that have undergone CreERT2‐mediated recombination and their wildtype counterparts simultaneously. In adult tail we observed dermal, epidermal and hair follicle‐localised melanocytes including populations in the hair matrix and occasionally the bulge and sebaceous glands (Figure 2e) labelled with H2B‐Cerulean. On tamoxifen induction we could label subpopulations with EYFP (Figure 2f,g). Our marcks‐mKate2 marker was easier to resolve by confocal imaging in adult skin than in embryonic skin and we could clearly see labelled dendrites (Figure 2g). We compared the recombination efficiency of the *R26R‐EYFP* reporter on administration of tamoxifen by gavage and intraperitoneal injection. We achieved the highest recombination efficiency using gavage for embryonic trunk melanoblasts and by using multiple IP injections for adult interfollicular melanocytes (Figure 2h,i).

**FIGURE 2:**
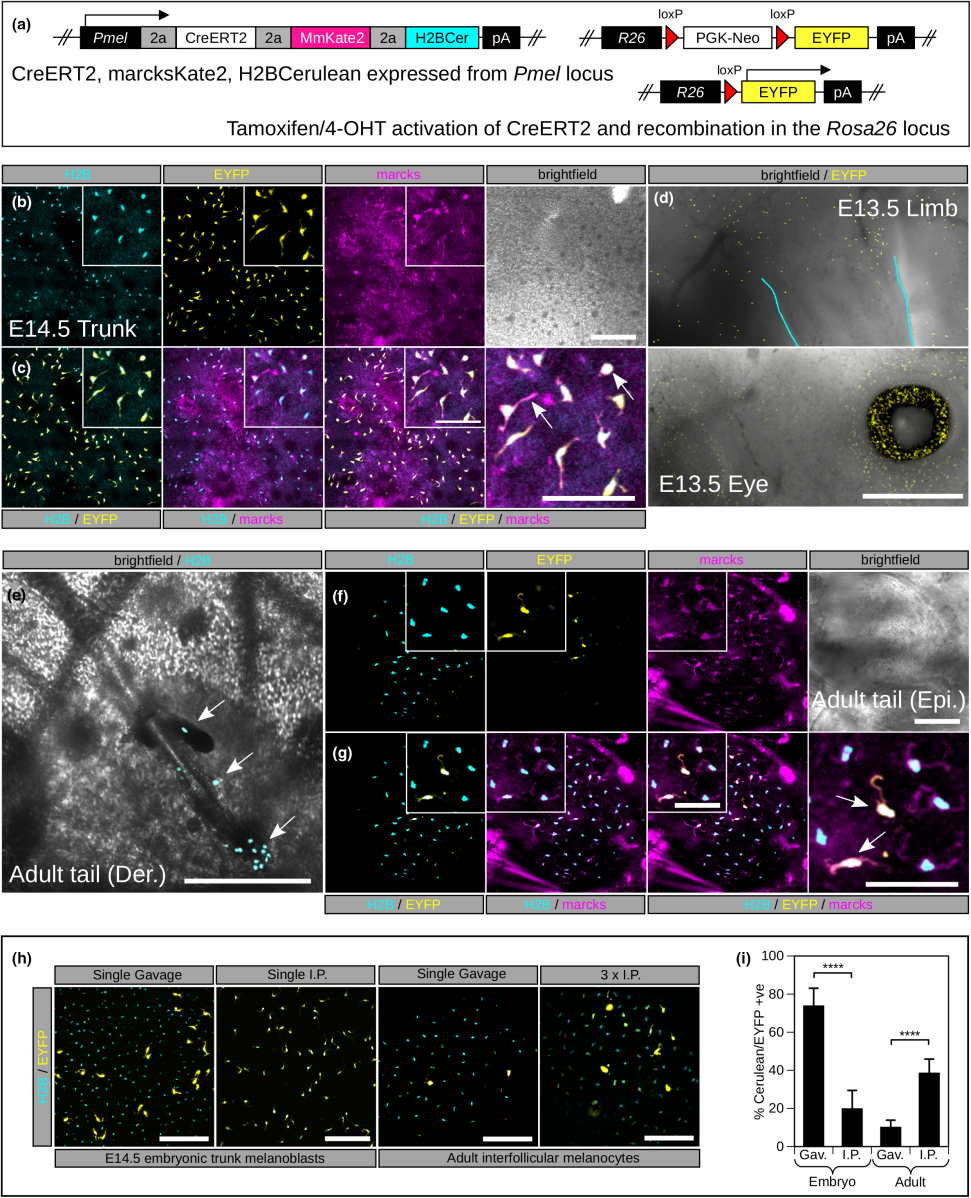
*Pmel‐CMN* expression in adult and embryonic mice and lineage tracing with *Rosa26* ^*EYFP*^. (a). Lineage tracing strategy. The CMN imaging cassette is expressed exclusively in melanoblasts/melanocytes coupled to endogenous *Pmel* expression. CreERT2 is activated by exposure to tamoxifen or 4‐OHT resulting in translocation to the nucleus and activation of the *R26R‐EYFP* reporter. (b). Expression of H2B‐Cerulean (nucleus), marcks‐mKate2 (membrane) and EYFP (cell body) in mouse trunk melanoblasts at E14.5 and (c) merged images showing the overlap in labelling from these probes. Pregnant *Pmel‐CMN; R26R‐EYFP* animals were administered tamoxifen by gavage. Examination of cell morphology across dermal‐epidermal Z‐stacks suggested that no labelling of nerves or other skin associated lineages was evident. The pseudopods of the migrating melanoblasts as well as mitotic cells were clearly observed (arrows in c). (d). EYFP labelled melanoblasts migrating towards the developing limb bud at E13.5 and expression of EYFP in the retinal pigmented epithelium of the eye. (e). Adult tail skin imaged from the dermal side – H2B‐Cerulean labelled melanoblasts were observed in the dermal papilla, bulge and occasional sebaceous glands (arrows in e). (f). Adult *Pmel* ^*CMN/CMN*^ *; Rosa26* ^*EYFP/EYFP*^ animals were given tamoxifen by injection and tail skin epidermis was imaged (g) merged images showing the overlap in labelling of the probes EYFP ‐ labelled a subset of epidermal melanocytes (arrows in g) marcks‐mKate2 localisation was easier to detect in adult than embryonic tissue. (h). Comparison of recombination efficiency in the *Rosa26* locus for administration of tamoxifen by gavage and intraperitoneal injection. (i). Quantification of the data in h. Epi, epidermis; Der, dermal. Scale bars: b = 200 μm; c (inset) = 100 μm; c = 100 μm; d = 1000 μm; e = 250 μm; f = 200 μm; g (inset) = 100 μm; g = 100 μm; h (embryo) = 200 μm; h (adult) = 100 μm; **** = Student's *t*‐test *p* < .0001.

Our experience with the *Dct::LacZ* (Mackenzie et al., 1997) and *Tyr::Cre* (Delmas et al., 2003) transgenic lines is that they tended to label the dorsal root ganglia (DRG) and underlying peripheral nerves in the dermis making imaging studies difficult and necessitating image processing to remove these aspects from our movies before we could perform cell tracking. Confocal tile scans encompassing the dorsal‐ventral aspect of the trunk did not reveal staining of the DRG by either the endogenously expressed H2B‐Cerulean marker or EYFPR marker in *Pmel* ^*CMN/CMN*^ *; Rosa26* ^*EYFP/EYFP*^ embryos (Figure 3a). We next compared our *Pmel* ^*CMN/CMN*^ *; Rosa26* ^*EYFP/EYFP*^ model to the previously published *Tyr::Cre;R26* ^*EYFP/EYFP*^ model (Delmas et al., 2003; Mort et al., 2010, 2016). We observed an extensive network of peripheral axons in the trunk dermis below the epidermal melanocyte population in *Tyr::Cre;R26* ^*EYFP/EYFP*^ animals, which were not apparent in the *Pmel* ^*CMN/CMN*^ *; Rosa26* ^*EYFP/EYFP*^ model either for the endogenously expressed H2B‐Cerulean marker or EYFPR (Figure 3b–e). Tracking of migrating cells in time lapse movies without the need for preprocessing from cultured *Pmel* ^*CMN/CMN*^ *; Rosa26* ^*EYFP/EYFP*^ embryonic skin at E14.5 showed classical undirected melanoblast behaviour as previously described (Mort et al., 2016). Next, to confirm cell‐type specificity we FACS sorted melanoblasts (Figure 3g), performed bulk RNA sequencing and examined the expression level in reads per kilobase of transcript, per million mapped reads (RPKM) for a panel of markers to distinguish; melanoblasts, keratinocytes, Schwann cell precursors (SCP), nerves/glia, muscle and fibroblasts (Figure 3h). We observed robust expression of the melanoblast markers *Kit, Gpnmb, Dct, Pmel, Tyr* and very low or undetectable levels of transcripts associated with other skin cell types including SCP's and peripheral nerves (Figure 3h). Transcript abundance for the grouped melanoblast markers shown in Figure 3h was at least 16‐fold higher than the abundance of the grouped markers for all other cell types. This difference was highly statistically significant in all cases (Figure 3i).

**FIGURE 3:**
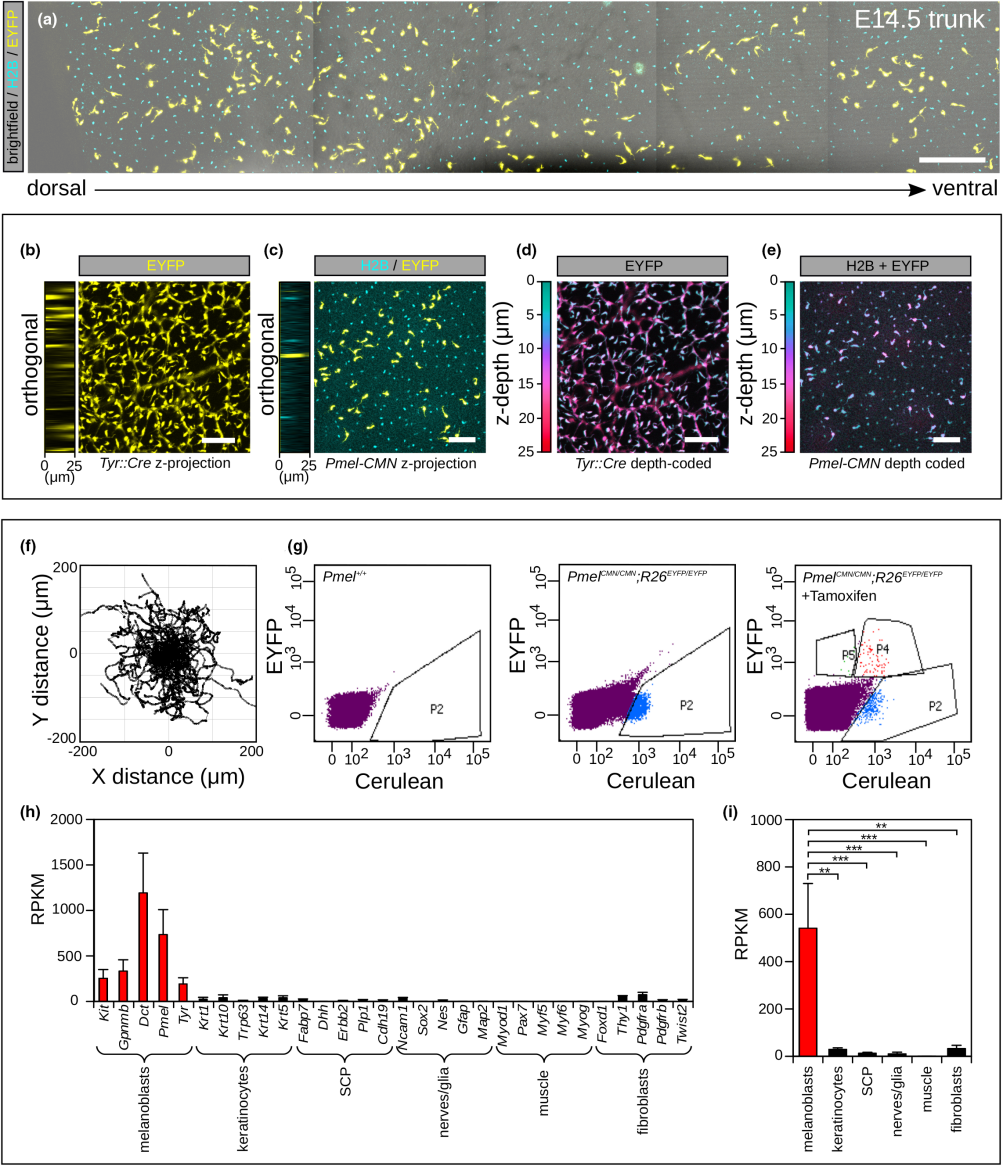
*Pmel‐CMN* activity is highly melanoblast specific in the developing embryo. (a). A confocal z‐projection of a tile scan spanning the dorsoventral axis of the embryonic trunk in an E14.5 *Pmel* ^*CMN/CMN*^;*R26* ^*EYFP/EYFP*^ embryo. EYFP was activated by tamoxifen injection at E10.5. A subpopulation of melanoblasts labelled with EYFP are present across the length of the DV‐axis. H2B‐Cerulean and EYFP were expressed cleanly in the migrating melanoblast population but not the surrounding keratinocytes or underlying nerves. (b–e). Comparison of labelling using *Tyr::Cre;R26* ^*EYFP/EYFP*^ (b,d) or *Pmel* ^*CMN/CMN*^;*R26* ^*EYFP/EYFP*^ (c,e). A 25 μm confocal stack was taken composed of sequential slices starting with the plane of the epidermal melanoblasts and finishing in the underlying epidermis. As well as labelling melanoblasts, EYFP expression can be observed in the underlying nerves in the dermis in *Tyr::Cre;R26* ^*EYFP/EYFP*^ skin but not *Pmel* ^*CMN/CMN*^;*R26* ^*EYFP/EYFP*^ skin as demonstrated by the z‐projection and orthogonal views (b,c) and the depth coded stacks (d,e). (f). Automated tracking of *Pmel* ^*CMN/CMN*^ *;R26* ^*EYFP/EYFP*^ labelled melanoblasts migrating in the developing trunk epidermis at E14.5 showing no preferred direction of migration. (g). Analysis of skin cells from *Pmel* ^*+/+*^ and *Pmel* ^*CMN/CMN*^ *;R26* ^*EYFP/EYFP*^ (± 4‐OHT) pooled litters after 4‐OHT injection at E11.5. A robust single labelled H2B‐Cerulean population (P2) is present in the absence of 4‐OHT. Robust single labelled H2B‐Cerulean (P2) and double labelled EYFP/H2B‐Cerulean (P4) populations are present after 4‐OHT induction. EYFP/H2B‐Cerulean (P) melanoblasts were FACS sorted for downstream RNAseq analysis (*n* = 3 litters). h. Expression level (mean ± SEM) in reads per kilobase of transcript, per million mapped reads (RPKM) for a panel of markers specific to: melanoblasts, keratinocytes, Schwann cell precursors (SCP), nerves/glia, muscle and fibroblasts. i. When grouped, the melanoblast specific transcripts shown in h were significantly over represented in the RNAseq results compared with the transcripts from all other cell types (mean ± SEM). One‐way analysis of variance (ANOVA) *p* < .001, pairwise Tukey's honestly significant difference test results indicated on the graph (** *p* < .01, *** *p* < .001). Scale bars: a = 250 μm; b = 100 μm. Orthogonal views in b and c are from epidermis (left = 0 μm) to the dermis (right = 25 μm); depth coding in d is from the epidermis (green = 0 μm) to the dermis (red = 25 μm).

In summary we describe *Pmel‐CMN* a multicistronic allele of *Pmel* that allows genetic modification and labelling of the melanocyte lineage through CreERT2 activity while simultaneously monitoring the activity of the endogenous *Pmel* promoter through expression of the fluorescent probes marcks‐mKate2 and H2B‐Cerulean. *Pmel‐CMN* is highly specific to developing melanoblasts and differentiated melanocytes. In melanoblasts our imaging studies and RNAseq demonstrate the utility of the model for live‐imaging studies and for the isolation of a population of pure melanoblasts with very little expression of lineage markers from earlier or adjacent populations. The *Pmel‐CMN* model will be useful for pigment cell biologists to dissect the embryonic origins of developing melanoblasts. It will be further useful for making melanocyte‐specific genetic manipulations in which both recombined and wildtype cells can be observed in the same time lapse video thus serving as an internal control. Furthermore, the general strategy for targeting the *Pmel* locus using a 2a self‐cleaving peptide will be useful to those wishing to drive other cDNAs in melanocytes in a transparent way.

## AUTHOR CONTRIBUTIONS

RLM and IJJ contributed to conceptualisation; ELW, RLM, LCB and ZCS did formal analysis; RLM, SLA and IJJ did funding acquisition; ELW, ZCS, LCB and RLM did investigation; RLM, MAK and PB contributed to methodology; ELW and RLM contributed to project administration; RLM and IJJ LMM contributed to resources; RLM contributed to software; RLM supervision; ELW and LCB contributed to validation; ELW, LCB and RLM contributed to visualisation; ELW and RLM contributed to writing – original draft preparation; ELW, LCB, KS, LMM and IJJ RLM contributed to writing, review and editing of the manuscript.

## CONFLICT OF INTEREST

The authors state that they have no conflict of interest.

## Supporting information


Appendix S1

Video S1

## Data Availability

The data that support the findings of this study are openly available in Pure at https://www.research.lancs.ac.uk/portal/en/people/richard‐mort(ed9f7471‐68c9‐4816‐97a9‐58f8f44c1f1dt).html.

## References

[pcmr13076-bib-0001] Adameyko, I. , Lallemend, F. , Aquino, J. B. , Pereira, J. A. , Topilko, P. , Müller, T. , Fritz, N. , Beljajeva, A. , Mochii, M. , Liste, I. , Usoskin, D. , Suter, U. , Birchmeier, C. , & Ernfors, P. (2009). Schwann cell precursors from nerve innervation are a cellular origin of melanocytes in skin. Cell , 139(2), 366–379. 10.1016/j.cell.2009.07.049 19837037

[pcmr13076-bib-0002] al‐Shawi, R. , Kinnaird, J. , Burke, J. , & Bishop, J. O. (1990). Expression of a foreign gene in a line of transgenic mice is modulated by a chromosomal position effect. Molecular and Cellular Biology, 10(3), 1192–1198. 10.1128/mcb.10.3.1192-1198.1990 2304463 PMC360995

[pcmr13076-bib-0003] Aydin, I. T. , & Beermann, F. (2011). A mart‐1::Cre transgenic line induces recombination in melanocytes and retinal pigment epithelium. Genesis (New York, N.Y.: 2000), 49(5), 403–409. 10.1002/dvg.20725 21309074

[pcmr13076-bib-0004] Baxter, L. L. , & Pavan, W. J. (2003). Pmel17 expression is Mitf‐dependent and reveals cranial melanoblast migration during murine development. Gene Expression Patterns: GEP, 3(6), 703–707. 14643677 10.1016/j.modgep.2003.07.002

[pcmr13076-bib-0005] Bosenberg, M. , Muthusamy, V. , Curley, D. P. , Wang, Z. , Hobbs, C. , Nelson, B. , Nogueira, C. , Horner, J. W. , Depinho, R. , & Chin, L. (2006). Characterization of melanocyte‐specific inducible Cre recombinase transgenic mice. Genesis (New York, N.Y.: 2000), 44(5), 262–267. 10.1002/dvg.20205 16676322

[pcmr13076-bib-0006] Davis, N. , Yoffe, C. , Raviv, S. , Antes, R. , Berger, J. , Holzmann, S. , Stoykova, A. , Overbeek, P. A. , Tamm, E. R. , & Ashery‐Padan, R. (2009). Pax6 dosage requirements in iris and ciliary body differentiation. Developmental Biology, 333(1), 132–142. 10.1016/j.ydbio.2009.06.023 19563798

[pcmr13076-bib-0007] Delmas, V. , Martinozzi, S. , Bourgeois, Y. , Holzenberger, M. , & Larue, L. (2003). Cre‐mediated recombination in the skin melanocyte lineage. Genesis (New York, N.Y.: 2000), 36(2), 73–80. 10.1002/gene.10197 12820167

[pcmr13076-bib-0008] Guyonneau, L. , Rossier, A. , Richard, C. , Hummler, E. , & Beermann, F. (2002). Expression of Cre recombinase in pigment cells. Pigment Cell Research, 15(4), 305–309. 10.1034/j.1600-0749.2002.02039.x 12100497

[pcmr13076-bib-0009] Li, A. , Ma, Y. , Yu, X. , Mort, R. L. , Lindsay, C. R. , Stevenson, D. , Strathdee, D. , Insall, R. H. , Chernoff, J. , Snapper, S. B. , Jackson, I. J. , Larue, L. , Sansom, O. J. , & Machesky, L. M. (2011). Rac1 drives Melanoblast organization during mouse development by orchestrating pseudopod‐ driven motility and cell‐cycle progression. Developmental Cell, 21(4), 722–734. 10.1016/j.devcel.2011.07.008 21924960 PMC3464460

[pcmr13076-bib-0010] Mackenzie, M. A. F. , Jordan, S. A. , Budd, P. S. , & Jackson, I. J. (1997). Activation of the receptor tyrosine kinase kit is required for the proliferation of Melanoblasts in the mouse embryo. Developmental Biology, 192(1), 99–107. 10.1006/dbio.1997.8738 9405100

[pcmr13076-bib-0011] Mort, R. L. , Hay, L. , & Jackson, I. J. (2010). Ex vivo live imaging of melanoblast migration in embryonic mouse skin. Pigment Cell and Melanoma Research, 23(2), 299–301. 10.1111/j.1755-148X.2010.00669.x 20067551 PMC2859249

[pcmr13076-bib-0012] Mort, R. L. , Jackson, I. J. , & Elizabeth Patton, E. (2015). The melanocyte lineage in development and disease. Development (Cambridge), 142(4), 620–632. 10.1242/dev.106567 PMC4325379 25670789

[pcmr13076-bib-0013] Mort, R. L. , Ross, R. J. H. , Hainey, K. J. , Harrison, O. J. , Keighren, M. A. , Landini, G. , Baker, R. E. , Painter, K. J. , Jackson, I. J. , & Yates, C. A. (2016). Reconciling diverse mammalian pigmentation patterns with a fundamental mathematical model. Nature Communications, 7, 10288. 10.1038/ncomms10288 PMC4729835 26732977

[pcmr13076-bib-0014] Rodríguez, C. I. , Buchholz, F. , Galloway, J. , Sequerra, R. , Kasper, J. , Ayala, R. , Stewart, A. F. , & Dymecki, S. M. (2000). High‐efficiency deleter mice show that FLPe is an alternative to Cre‐loxP. Nature Genetics, 25(2), 139–140. 10.1038/75973 10835623

[pcmr13076-bib-0015] Srinivas, S. , Watanabe, T. , Lin, C.‐S. , William, C. M. , Tanabe, Y. , Jessell, T. M. , & Costantini, F. (2001). Cre reporter strains produced by targeted insertion of EYFP and ECFP into the ROSA26 locus. BMC Developmental Biology, 1, 4. 10.1186/1471-213X-1-44 11299042 PMC31338

[pcmr13076-bib-0016] Tonks, I. D. , Nurcombe, V. , Paterson, C. , Zournazi, A. , Prather, C. , Mould, A. W. , & Kay, G. F. (2003). Tyrosinase‐Cre mice for tissue‐specific gene ablation in neural crest and neuroepithelial‐derived tissues. Genesis (New York, N.Y.: 2000), 37(3), 131–138. 10.1002/gene.10242 14595836

[pcmr13076-bib-0017] Viros, A. , Sanchez‐Laorden, B. , Pedersen, M. , Furney, S. J. , Rae, J. , Hogan, K. , Ejiama, S. , Girotti, M. R. , Cook, M. , Dhomen, N. , & Marais, R. (2014). Ultraviolet radiation accelerates BRAF‐driven melanomagenesis by targeting TP53. Nature, 511(7510), 478–482. 10.1038/nature13298 24919155 PMC4112218

[pcmr13076-bib-0018] Wilson, C. , Bellen, H. J. , & Gehring, W. J. (1990). Position effects on eukaryotic gene expression. Annual Review of Cell Biology, 6, 679–714. 10.1146/annurev.cb.06.110190.003335 2275824

[pcmr13076-bib-0019] Yajima, I. , Belloir, E. , Bourgeois, Y. , Kumasaka, M. , Delmas, V. , & Larue, L. (2006). Spatiotemporal gene control by the Cre‐ERT2 system in melanocytes. Genesis (New York, N.Y.: 2000), 44(1), 34–43. 10.1002/gene.20182 16419042

