# Supplementary material for: Genetically engineered multicistronic allele of Pmel yielding highly specific CreERT2‐mediated recombination in the melanocyte lineage
Source: Pigment Cell Melanoma Res. 2022 Dec 19;36(1):71–7. doi: 10.1111/pcmr.13076 (PMC10107733; doi:10.1111/pcmr.13076)
Supplement: Supplementary file 1 — Appendix S1 [file PCMR-36-71-s001.pdf]

Genetically engineered multicistronic allele of *Pmel* yielding highly specific CreERT2 mediated recombination in the melanocyte lineage

Authors:

Emma L. Wilkinson[1*], Louise C. Brennan[1*], Olivia J. Harrison[2], Zoe Crane-Smith[2], Philippe Gautier[2], Margaret A. Keighren[2], Peter Budd[2], Karthic Swaminathan[3], Laura M. Machesky[4], Sarah L. Allinson[1], Ian J. Jackson[2,5] and Richard L. Mort[1]

Contact Information:

1. Division of Biomedical and Life Sciences, Faculty of Health and Medicine, Lancaster University, Lancaster, UK.

2. MRC Human Genetics Unit, Institute of Genetics and Cancer, Western General Hospital, University of Edinburgh, EH4 2XU, UK.

3. Centre for Skin Sciences, Faculty of Life Sciences, University of Bradford, Bradford, BD7 1DP, UK.

4. Cancer Research UK, Beatson Institute, and Institute of Cancer Sciences, University of Glasgow, Glasgow, UK.

5. Roslin Institute, University of Edinburgh, Roslin, EH25 9RG, UK.

Corresponding author: r.mort@lancaster.ac.uk

*These authors contributed equally to the work.

SUPPORTING INFORMATION:

Movie S1: Expression of H2B-Cerulean (nucleus), marcks-mKate2 (membrane) and EYFP (cell body) in mouse trunk melanoblasts at E14.5. Pregnant *Pmel-CMN; R26R-EYFP* animals were administered Tamoxifen by gavage at E10.5. The dendrites and pseudopods of the migrating melanoblasts as well as mitotic cells can be clearly observed as the time lapse sequence progresses. Scale bar = 100 µm.

MATERIALS AND METHODS

Animal work

All animal work was approved by a University of Edinburgh and/or Lancaster University internal ethics committee and was performed in accordance with institutional guidelines under licence by the UK Home Office (PPL 60/4424, PPL 60/3785 and PPL P4FF1DB05). Mice were maintained in the animal facilities of the University of Edinburgh and Lancaster University. *Pmel-CMN* mice were maintained on a C57BL/6J background. Mice were genotyped using PCR as detailed below, they were housed in a barrier facility with 12-hour light and dark cycles. Tamoxifen (Sigma) was dissolved in corn oil and administered by oral gavage to pregnant dams at E11.5 at 8 mg per 40g body weight. 4-OHT (Sigma) was dissolved in corn oil and administered by intraperitoneal injection at 8 mg per 40g body weight.

Genotyping

Pmel-CMN mice were genotyped by PCR (35 cycles: denaturation - 94 ℃ for 30 secs; annealing - 60 ℃ for 30 secs; extension - 72 ℃ for 30 secs) using the primers WT2_For and WT2_Rev to amplify a 568 bp wildtype and WT2_For and CMN_Rev to amplify a 354 bp targeted band (Table 3). *R26R-EYFP* mice were genotyped by PCR (35 cycles: denaturation - 94 ℃ for 30 secs; annealing - 58 ℃ for 30 secs; extension - 72 ℃ for 30 secs) using the primers R26_For and R26_Rev to amplify a 237 bp wildtype band and SA_For and SA_Rev to amplify a 320 bp targeted band (Table 3). *Tyr::Cre* mice were genotyped by PCR (35 cycles: denaturation - 94 ℃ for 15 secs; annealing - 62 ℃ for 30 secs; extension - 72 ℃ for 30 secs) using the primers TyrCre_F and TyrCre_R to amplify a 473 bp band corresponding to the *Tyr::Cre* transgene (Table 3). *FLPe* mice mice were genotyped by PCR (35 cycles: denaturation - 94 ℃ for 15 secs; annealing - 66 ℃ for 30 secs; extension - 72 ℃ for 15 secs) using the primers FLPe_F and FLPe_R to amplify a 145 bp band corresponding to the *FLPe* transgene (Table 3).

All reactions were carried out using Taq polymerase (Thermo Fisher Scientific) in 1 X reaction buffer supplemented with 1.5mM $MgCl_2$ according to the manufacturer's instructions.

Assembly of *Pmel* targeting construct

A 191 bp fragment containing the coding sequence for the myristoylated alanine-rich C-kinase substrate (marcks) was amplified from ROSA26-mTmG (Muzumdar et al., 2007) as template (a gift from Liqun Luo - Addgene plasmid # 17787 ; http://n2t.net/addgene:17787 ; RRID:Addgene_17787) by PCR using the primers marcks_F and marcks_R (Table 1) and subcloned into pmKate2-N (Evrogen) as a EcoRI/BsrGI fragment. Cerulean (Rizzo et al., 2004) was amplified from Cerulean (a gift from Dave Piston - Addgene plasmid # 15214 ; http://n2t.net/addgene:15214 ; RRID:Addgene_15214) by PCR using the primers Cer_F and Cer_R (Table 1). The Cerulean PCR product was cut with KpnI, blunted, and cut with AgeI. In parallel pH2B-GFP (Kanda et al., 1998) plasmid (a gift from Geoff Wahl - Addgene plasmid # 11680 ; http://n2t.net/addgene:11680 ; RRID:Addgene_11680) was cut with NotI, blunted, and cut with AgeI allowing subcloning of Cerulean into pH2B-GFP as an AgeI-blunt fragment replacing GFP. Marcks-mKate2 and H2B-Cerulean were subsequently fused by overlap extension PCR incorporating the foot and mouth disease virus 2a peptide sequence between each fusion gene using the primers marcks2_F, Cer2_R, H2B_t2a_F and mKate_t2a_R (Table 1). The subsequent PCR product was cloned into pCAGiP (a gift from Ian Chambers) as a PacI/XhoI fragment resulting in pCAG-MN-iP. CreERT2 was amplified from pCreERT2 (a gift from Laura Lettice) using the primers CreERT2_F and CreERT2_R and (Table 1) and cloned into pCAG-MN-IP as a MluI/MfeI fragment resulting in pCAG-CMN-iP. The *Pmel* targeting construct was assembled using pCAGiP as backbone. *Pmel* Exon 11 was PCR amplified using Pmel_Exon11_F and Pmel_Exon_11_R to remove the stop codon and add an f2a sequence to the end of the exon. This fragment was then cloned into pCAGiP as a SalI-MluI fragment to yield pPmel-Exon11. A 5’ homology arm was subsequently PCR amplified with the primers Pmel_5_Hom_F and Pmel_5_Hom_R and cloned into pExon11 as a SalI/FseI fragment to yield pPmel-5-Exon11. A 3’ homology arm was then amplified with the primers Pmel_3_Hom_F and Pmel_3_Hom_R and cloned as an AscI fragment to yield pPmel-5-Exon11-3. Finally a pA-frt-PGK-Neo-pA-frt cassette ROSA26-mTmG (Addgene #17787) was cloned 3’ of Exon11 as KpnI/AscI fragment followed by cloning of the CMN cassette from pCAG-CMN-iP as a MluI/KpnI fragment to yield pPmel-CMN.

ES Cell targeting and generation of transgenic mice

E14 ES cells were maintained in Glasgow Minimum Essential Medium (GMEM) supplemented with 10% fetal calf serum, 0.1mM non-essential amino acids, 2mM L-Glutamine, 1mM sodium pyruvate, 0.1mM β-mercaptoethanol and 106 units/L LIF. They were electroporated with linearized pPmel-CMN plasmid using standard procedures. Clones were picked after 14 days of G418 selection. Screening across the *Pmel* 5′ homology arm (35 cycles: denaturation - 98 °C for 10 secs; annealing - 66 °C for 10 secs; extension - 72 °C for 30 secs) was performed using the primers Pmel_5_For and Pmel_5_Rev to generate a 5.7 kb targeted band (Table 2). A second control PCR (35 cycles: denaturation - 98 °C for 10 secs; annealing - 60 °C for 10 secs; extension - 72 °C for 30 secs) was conducted to demonstrate DNA integrity using the primers Pmel_Wt_For and Pmel_Wt_Rev to generate a 2.4 kb wild type band from the *Pmel* locus. Correct targeting was confirmed on the positive clones by PCR amplification (35 cycles: denaturation - 98 °C for 10 secs; annealing - 68 °C for 10 secs; extension - 72 °C for 30 secs) of a 2.5 kb targeted band across the *Pmel* 3′ homology arm using the primers Pmel_3_For and Pmel_3_Rev (Table 2) to amplify a 2.5 kb targeted band. All PCR reactions were carried out using 50 ng genomic DNA using Phusion Hotstart II DNA polymerase (Thermo Fisher Scientific) with GC buffer according to the manufacturers standard reaction conditions. Transgenic mice were produced by blastocyst injection of *Pmel-CMN* ES cells according to standard methods using C57BL/6J mice. Germline transmission was identified after a single round of ES cell blastocyst injections. Subsequent intercrosses generated $Pmel^{+/+}$, $Pmel^{+/CMN}$ and $Pmel^{CMN/CMN}$ offspring at near Mendelian ratios.

RNA sequencing

Dissected skin samples were pooled from whole litters of *Pmel-CMN;R26R-EYFPR* litters at E13.5-14.5 and incubated at room temperature in 10 mM v/v EDTA (Sigma-Aldrich) in PBS for 30-45 mins followed by 30 mins in Liberase (0.2 Wünsch U/ml in DMEM) at 37°C. To prepare a single cell suspension, cells were taken through a series of hypodermic needles from 19G-25G followed by a 40 μm cell strainer (Corning). Samples were centrifuged, and resuspended in PBS before FACS sorting using an Aria2 SORP cell sorter (Becton Dickinson) directly into 1.5 ml tubes (Eppendorf) containing 350 μl of RLT buffer. cDNA was synthesised using the Ovation RNA-Seq System V2 (NuGen) following the manufacturer's instructions. RNA sequencing analysis was performed in R using standard analysis tools and packages. Briefly, data quality was assessed using FastQC (Andrews, 2010). RNA-Seq reads were aligned to the mouse

reference genome using Tophat (Trapnell et al., 2009) which in turn uses the high-throughput read aligner Bowtie (Langmead et al., 2009). Read count analysis was performed using DESeq (Anders & Huber, 2010). Reads per kilobase of transcript, per million mapped reads (RPMK) values were then calculated using Microsoft Excel followed by the mean RPMK value for each gene (n = 3 repeats).

Cell culture

HeLa cells were maintained in Dulbecco's modified Eagle's medium (DMEM) containing 0.11 g/liter sodium pyruvate, 2 mM l-glutamine, 4.5 g/liter glucose, 10% fetal bovine serum (FBS), 100 U/ml penicillin, and 100 U/ml streptomycin in a humidified incubator at 5% (v/v) CO_2. They were transfected with pCAG-CMN-iP using Lipofectamine 2000 (Thermo Fisher) according to the manufacturer's instructions.

Fluorescence microscopy and live Imaging

Imaging of embryonic and adult skin was performed on either a Nikon A1R confocal microscope or Zeiss 880 confocal microscope surrounded by an environmental chamber providing 5% CO_2 in air and maintained at a constant stage top temperature of 37 ℃. Embryonic skin culture was performed according to the method described in Mort et al (2010).

Image Analysis

Image analysis tasks were performed with the Fiji (Schindelin et al., 2012) distribution of ImageJ an open source image analysis package based on NIH Image (Schneider et al., 2012). The depth coded stacks and orthogonal views presented in Figure 3 were prepared from 25 μm confocal stacks composed of sequential slices starting at the plane of the epidermal melanoblasts and finishing in the underlying epidermis. They were assembled using the ‘Orthogonal Views’ command. The depth coded stacks were generated so that the colour of a pixel represented the z-depth from the epidermis using the ‘Temporal-Color Code’ command.

TABLES

Table 1: Oligonucleotide sequences used for cloning:

**Name**	**Sequence (5′ - 3′)**
**Oligonucleotide sequences used for cloning (additional features in brackets/underlined):**	
marcks_F (EcoRI)	CGG CAC GAA TTC ATG GGT TGC TGT TTC TCC AAG
marcks_R (BsrGI)	GCC GTC TGT ACA GCT TCA TGT GCA TGT TCT CCT TAA TCA GCT CGC TCA CGG ATC CTA CCT TCA CGT GGC
Cer_F (KpnI)	CGG CAC GGT ACC ATG GTG AGC AAG GGC GAG G
Cer_Rev (AgeI)	GCC GTC ACC GGT TTA CTT GTA CAG CTC GTC CAT G
marcks2_F (PacI, MfeI)	CGG CAC TTA ATT AAC AAT TG ATG GGT TGC TGT TTC TCC AAG AC
Cer2_R (XhoI)	GCC GTC CTC GAG CGG CCG CGG TAC CTT ACT TGT ACA GCT CGT CCA TG
H2B_t2a_F (t2a)	GGT AGC GGT GAA GGC CGT GGC TCT CTT CTC ACT TGT GGC GAT GTC GAA GAG AAT CCT GGA CCC ATG CCA GAG CCA GCG AAG TCT GCT C
mKate_t2a_R (t2a)	GGGTCCAGGATTCTCTTCGACATCGCCACAAGTGAGAAGAGAGCCACGGCCTTCACCGCTACCTCTGTGCCCCAGTTTGCTAGG
CreERT2_F (MluI)	CGG CAC ACG CGT A TGT CCA ATT TAC TGAC
CreERT2_R (MfeI, f2a)	GCC GTC CAA TTG TGG ACC TGG ATT GCT TTC TAC ATC CCC AGC CAG TTT GAG TAA ATC AAA GTT AAG AGT TTG TTT GAC AGC TGT GGC AGG GAA ACC CTC
PmeI_5_Hom_F (SalI)	CGG CAC GTC GAC GAG CAT ACC CGG AGG AAT GAC AGA GCT TG
PmeI_5_Hom_R (FseI)	GCC GTC GGC CGG CCC ACT GGC CTC GGA CTC ACT C
PmeI_3_Hom_F (AscI)	CGG CAC GGC GCG CCC AGA ACC TTG CGG GAG GGC
PmeI_3_Hom_R (MluI)	GCC GTC ACG CGT GTT ACC AAG CTT GAT AGC CTG
PmeI_Exon_11_F (SalI, FseI)	CGG CAC GTC GAC GCC GTC CGG CAC GGC CGG CCC TGA CCT CCA CAT GTG GAC C

Pmel_Exon_11_R (MluI, f2a)	GCC GTC ACG CGT CAG GAT TCT CTT CGA CAT CGC CAC AAG TGA GAA GAG AGC CAC GGC CTT CAC CGC TAC CGA CCT GCT GTC CAC TGA GGA G

Table 2: Oligonucleotide sequences used for screening ES cells:

**Name**	**Sequence (5′ - 3′)**
**Oligonucleotide sequences used for screening ES cells:**	
**Wildtype PCR (2.4 kb):**	
Pmel_Wt_For	CAG AAC CTT GCG GGA GGG C
Pmel _Wt_Rev	GCT GAC GCA GAA TGA AGA ACA AGC
**Long range PCR across 5’ Homology arm (5.7 kb):**	
Pmel_5_For	GCC TAC ACT GGT TGG TGC AAA TGC
Pmel_5_Rev	GGT GTA CGG TCA GTA AAT TGG ACA T
**Long range PCR across 3’ Homology arm (2.5 kb):**	
Pmel_3_For	GCT CGA CTA GAG CTT GCG GAA CC
Pmel_3_Rev	GCC ACA TCT GAG TCC ATC TTC TC

Table 3: Oligonucleotide sequences used for genotyping:

Name	Sequence (5′ - 3′)
**Oligonucleotide sequences used for genotyping *Pmel-CMN* mouse line (Wt = 568 bp, Mutant = 354 bp):**	
Pmel_Wt2_For	GGG TAA AGA AGA GGG GAG AGG
Pmel_Wt2_Rev	GGG ATG TTC CAT CAC CTT CA
Pmel_CMN_Rev	AGG CAA ATT TTG GTG TAC GG
**Oligonucleotide sequences used for genotyping *R26R-EYFP* mouse line (Wt = 237 bp, Mutant = 320 bp):**	
R26_For	TGT TAT CAG TAA GGG AGC T
R26_Rev	CAC ACC AGG TTA GCC TTT A
SA_For	AAA GTC GCT CTG AGT TGT TAT
SA_Rev	AAG ACC GCG AAG AGT TTG TC
**Oligonucleotide sequences used for genotyping *Tyr::Cre* mouse line (473 bp):**	
TyrCre_F	GTC ACT CCA GGG GTT GCT GG
TyrCre_R	CCG CCG CAT AAC CAG TGA
**Oligonucleotide sequences used for genotyping *FLPe* mouse line (145 bp):**	
FLPe_F	GTA AAA CAC CAC CTA AGG TG
FLPe_R	CTC AGC GAA TTG CTT ATG ATA G

REFERENCES:

Anders, S., & Huber, W. (2010). Differential expression analysis for sequence count data. *Genome Biology*, *11*(10), R106. https://doi.org/10.1186/gb-2010-11-10-r106

Andrews, S. (2010). *FastQC A Quality Control tool for High Throughput Sequence Data.* https://www.bioinformatics.babraham.ac.uk/projects/fastqc/

Kanda, T., Sullivan, K. F., & Wahl, G. M. (1998). Histone-GFP fusion protein enables sensitive analysis of chromosome dynamics in living mammalian cells. *Current Biology: CB*, *8*(7), 377–385. https://doi.org/10.1016/s0960-9822(98)70156-3

Langmead, B., Trapnell, C., Pop, M., & Salzberg, S. L. (2009). Ultrafast and memory-efficient alignment of short DNA sequences to the human genome. *Genome Biology*, *10*(3), R25. https://doi.org/10.1186/gb-2009-10-3-r25

Mort, R. L., Hay, L., & Jackson, I. J. (2010). Ex vivo live imaging of melanoblast migration in embryonic mouse skin. *Pigment Cell and Melanoma Research*, *23*(2). https://doi.org/10.1111/j.1755-148X.2010.00669.x

Muzumdar, M. D., Tasic, B., Miyamichi, K., Li, L., & Luo, L. (2007). A global double-fluorescent Cre reporter mouse. *Genesis (New York, N.Y. : 2000)*, *45*(9), 593–605. https://doi.org/10.1002/dvg.20335

Rizzo, M. a, Springer, G. H., Granada, B., & Piston, D. W. (2004). An improved cyan fluorescent protein variant useful for FRET. *Nature Biotechnology*, *22*(4), 445–449. https://doi.org/10.1038/nbt945

Schindelin, J., Arganda-Carreras, I., Frise, E., Kaynig, V., Longair, M., Pietzsch, T., Preibisch, S., Rueden, C., Saalfeld, S., Schmid, B., Tinevez, J.-Y., White, D. J., Hartenstein, V., Eliceiri, K., Tomancak, P., & Cardona, A. (2012). Fiji: An open-source platform for biological-image analysis. *Nature Methods*, *9*(7), 676–682. https://doi.org/10.1038/nmeth.2019

Schneider, C. A., Rasband, W. S., & Eliceiri, K. W. (2012). NIH Image to ImageJ: 25 years of image analysis. *Nature Methods*, *9*(7), 671–675. https://doi.org/10.1038/nmeth.2089

Trapnell, C., Pachter, L., & Salzberg, S. L. (2009). TopHat: Discovering splice junctions with RNA-Seq. *Bioinformatics (Oxford, England)*, *25*(9), 1105–1111. https://doi.org/10.1093/bioinformatics/btp120
